# Aeromagnetic and digital elevation model constraints on the structural framework of southern margin of the Middle Niger Basin, Nigeria

**DOI:** 10.1038/s41598-021-00829-y

**Published:** 2021-11-04

**Authors:** Naheem Banji Salawu

**Affiliations:** 1 BS Geophysical and Consultancy Ltd., Ilorin, Nigeria; 2 Department of Geophysics, Federal University Oye-Ekiti, Oye-Ekiti, Nigeria

**Keywords:** Geology, Geomagnetism, Geomorphology, Geophysics, Mineralogy, Tectonics

## Abstract

High resolution aeromagnetic anomaly and topographic data using enhancement filtering techniques have resulted in better understanding of the nature of structures at the margins of the Middle Niger Basin. Due to the lack of structural data, the gold deposits within the margins of the Middle Niger Basin were variably excavated, leading to land degradation. The newly-produced structural map highly constrained the mineralization, which makes the study area and its periphery safe for gold exploration. Structural map has been produced from the integration of derivative maps to assess the pattern of surface and subsurface structural features within the studied area. The structural map unravels structural features with major and minor structural trends, in addition to a prominent crustal partitioning, characterized by the Ifewara shear zone. These structural features correlate very well with known gold mineralized locations and also predict new zones for structurally controlled gold and associated mineralization. The structural patterns are directly related with tectonic episodes in the basement. In fact, the Ifewara shear zone appears more pronounced on the total gradient and 3-D Euler deconvolution maps, which reveal various lineaments within the shear zone. The topographic maps (digital elevation model) clearly shows the surface morphology of the region under study while the resulting shaded-relief map reveal the continuity of the deduced magnetic lineaments that coincide with the valleys of River Niger which exploits zones of weakness from the lineaments. The results suggest high sensitivity of fluvial network to deduced lineaments with possible significant implication for alluvial gold mineralization.

## Introduction

The constant demand for gold has resulted in the emergence and growing rate of artisanal mining activities around the boundary of the Middle Niger Basin. The miners in their bid to located gold mineralization has resulted to random excavation of potential gold sites leading to obvious loss of land for agricultural purposes^1^. The southern border of the Middle Niger Basin is known to host gold mineralization which occurs with quartz veins that are associated with structural features within the northern extension of the Ife-Ilesha schist belt. The sedimentary piles of the basin (northern parts of the study area) overlying the basement mask these structural features. Hence, any significant and economical gold mining exploitation will therefore be associated with the basement terrain south of the studied region. The gold mineralization within the study area is localized by lineament systems, which are hampered by lack of structural data. The aim is to differentiate basement lineaments from sedimentary basin structures and infer regions which are viable for exploitation of gold mineralization. It is worth to investigate these lineaments in order to produce a detailed structural map of the region, which would be useful for localizing new mining targets within the region. The accessibility of lineament systems of the study area are greatly reduced due to dense vegetation in a sub-tropical and tropical region such as Nigeria. This makes high resolution aeromagnetic and satellite imaging techniques more appropriate for the region.

Aeromagnetic survey and remote sensing methods are standard exploration techniques, which are highly suitable for mineral exploration programs^1,2^. Recent improvements in digital processing of remotely sensed imagery have led to higher resolution of delineation procedure of regional surface features. Similarly, significant improvements have also been made in the processing of aeromagnetic anomaly maps and in their integration with other data for optimum geological interpretation. Understanding if the anomalies in this context originate from bedrock or sedimentary units is essential for assessing basin structural features and depth to shallow mineral exploration targets^3^.

Aeromagnetic data also offer 3-Dimensional information about geological terrains as well as the plunge and dip of geological features through evaluation of gradients in the data^4^. The analysis and interpretation of aeromagnetic data via derivative techniques offer a direct means of revealing hidden tectonic structural features that host economical hydrocarbon and mineral resources. Hence, derivative techniques such as the total gradient^5–7^, horizontal gradient magnitude^8,9^ and first vertical derivative^10^ are regularly used for the interpretation of aeromagnetic data to obtain required information for hydrocarbon and mineral exploration^11,12^.

Structural interpretation requires multi-disciplinary techniques utilizing various datasets, which can be integrated with each other to produce a reliable complete interpretation. This study examines surface and subsurface configuration of southern border of the Middle Niger Basin using combined geological analysis of shuttle radar topography mission digital elevation model and aeromagnetic data. This is to provide the structural map of the region which can help provide location for structurally controlled mineralization. This information is significant for mineral investigations within the studied region.

## Geologic setting

**Figure 1:**
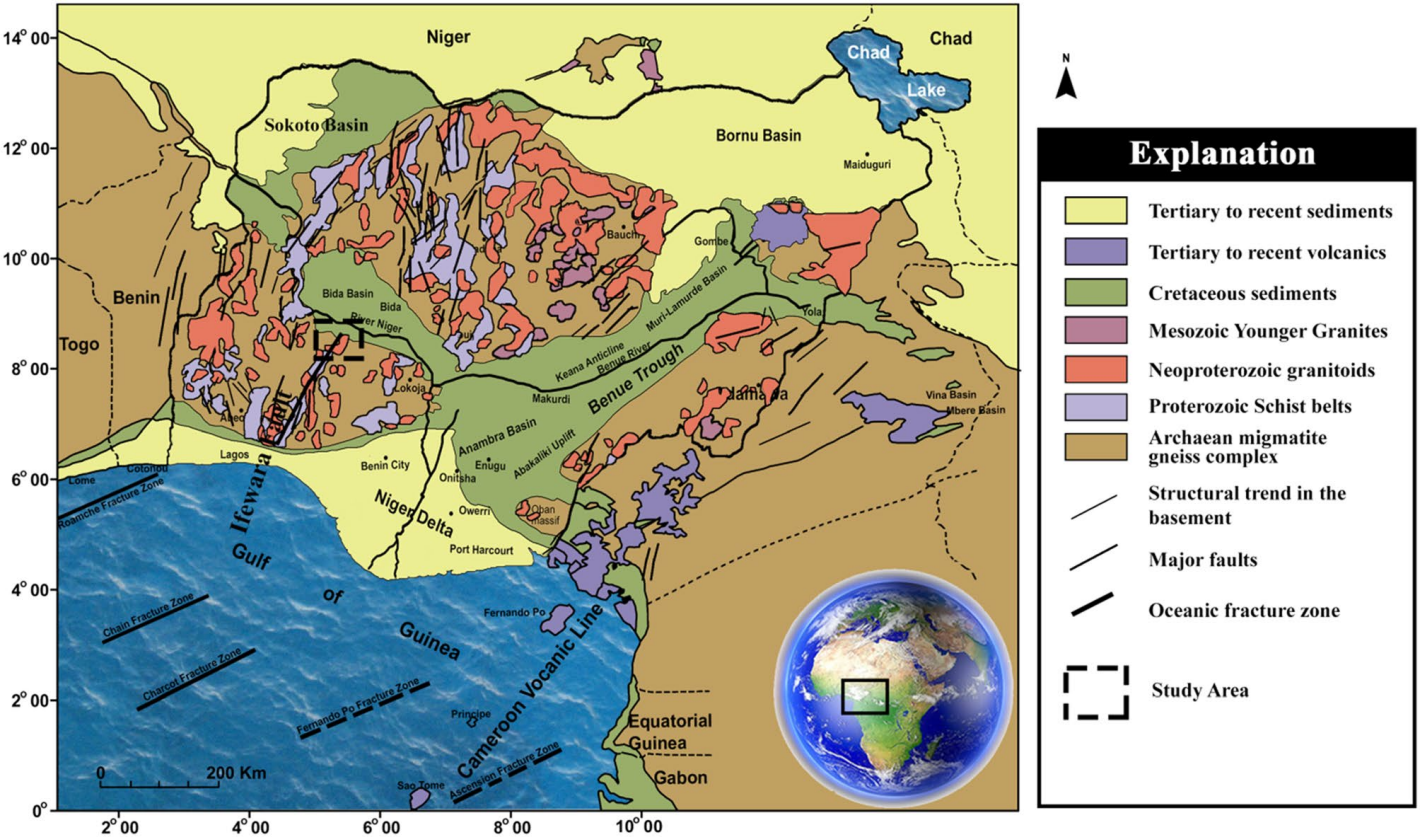
Generalized geological map of Nigeria and environs, showing the area were this study was conducted as dashed black lines. Note the Ifewara fault on the map as a thick black line. (Modified after Ref.^23^). Reprinted by permission from [the Licensor]: [Springer Nature] [Geomechanics and Geophysics for Geo-Energy and Geo-Resources] [Structural geometry of Ikogosi warm spring, southwestern Nigeria: evidence from aeromagnetic and remote sensing interpretation, Salawu et al.^15^ [COPYRIGHT] (2021).

**Figure 2:**
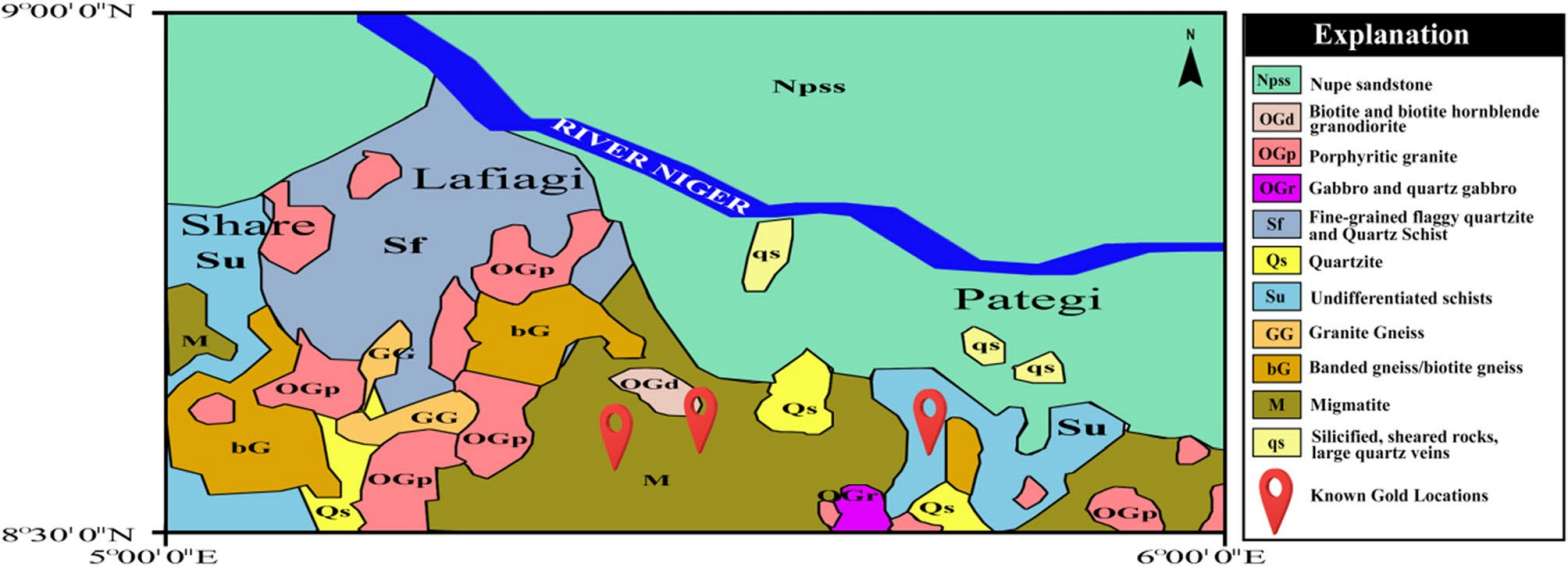
Simplified geological map of Lafiagi and Pategi regions^24^. Known gold locations adopted from Ref.^25^. The figure was produced using Adobe Photoshop CS5 Software version 12.0 (Website: https://www.adobe.com/products/photoshop.html).

The study area is shown as thick black lines on the generalized geological map of Nigeria in Fig. 1. The studied region (Lafiagi-pategi axis) is located within the boundary between the Middle Niger Basin and the Precambrian basement complex of Nigeria (Fig. 2). The rock types found in the northern part of the study area are predominantly Campano-Maastrichtian sandstones, while the southern part consists of metasedimentary rocks infolded into the migmatite-gneiss complex (Fig. 2). The Nigerian basement, including the entire study area witnesses the Pan-African Orogeny^13^. The impact of the Pan-African Orogeny (600 Ma) are mainly reworking of the polycyclic older crust, which gives dates that corresponds to the Liberian (2700 Ma), Eburnean (2000 Ma) and Kibaran (1100 Ma) orogenies^14^. Pre-Pan-African (probably Eburnean and Kibaran orogenies) structural features have been identified by Ref.^15^, within the southern part (Ikole/Kabba region) of the study area. The effect of Eburnean Orogeny on the Nigerian basement was probably accompanied by folding metamorphism, deformation, and igneous activities^16^. While the Eburnean Orogeny was main granite-forming tectonic event, the Kibaran Orogeny in Nigeria is largely ensialic tectonic event (though with probably small formation of ocean basin) which led into the further widespread and intense Pan-African Orogeny^14^. The Pan-African Orogeny produced heterogeneous reworking of the Nigerian terrain through widespread deformation, metamorphism and intrusion of granitoids, together with the generation of the dominant N–S and NE–SW shears such as the Kalangai-Zungeru-Ifewara shear zone^17^. The Kalangai-Zungeru-Ifewara shear zone extends from the basement terrain of western Nigeria through the basement of the Middle Niger Basin^1^. The Middle Niger Basin is an inland sedimentary basin situated in the west central part of Nigeria. The basin has an average sedimentary pile of approximately 950 m from recent aeromagnetic results of Refs.^1,18^ with two geographic subdivisions; the northern and southern segments. Four stratigraphic horizons in the northern part have been identified, which they include; the basal Bida Formation (conglomerate, sandstone) and Sakpe Ironstone of Campanian age and Enagi Formation (sandstone, siltstone, claystone) and Batati Ironstone of Maastrichtian age^19–21^. Their lateral southern equivalents are Lokoja Formation (conglomerate, sandstone) and Patti Formation (sandstone, shale, claystone) of Campanian age and the ferruginous Agbaja Formation of Maastrichtian age. The geological map of Lafiagi and Pategi region (Fig. 2) shows three locations of gold mineralization within the region. These gold mineralization locations will serve as control, calibration and validation of lineaments associated with gold deposits in subsequent structural map produced herein. Gold mineralization occurs with sulfides within quartz associated with extensive lineament system within the study area. Delineating these quartz veins is important and ideal for successful mineral exploration activities. The NNE–SSW trending Ifewara shear zone represents the main tectonic feature the region. The Ifewara shear zone is the southern segment of the NNE-trending Kalangai-Zungeru-Ifewara shear zone associated with gold mineralization^17,22^. The subsurface expression of the Ifewara shear zone is yet to be established within the study area. Hence, the study attempts to delineate structural features of the study area including the Ifewara shear zone by integrating aeromagnetic and topographic data. This is to provide important information about the geometry of structures associated with mineralization around the southern border of the Middle Niger Basin.

## Results and interpretation

**Figure 3:**
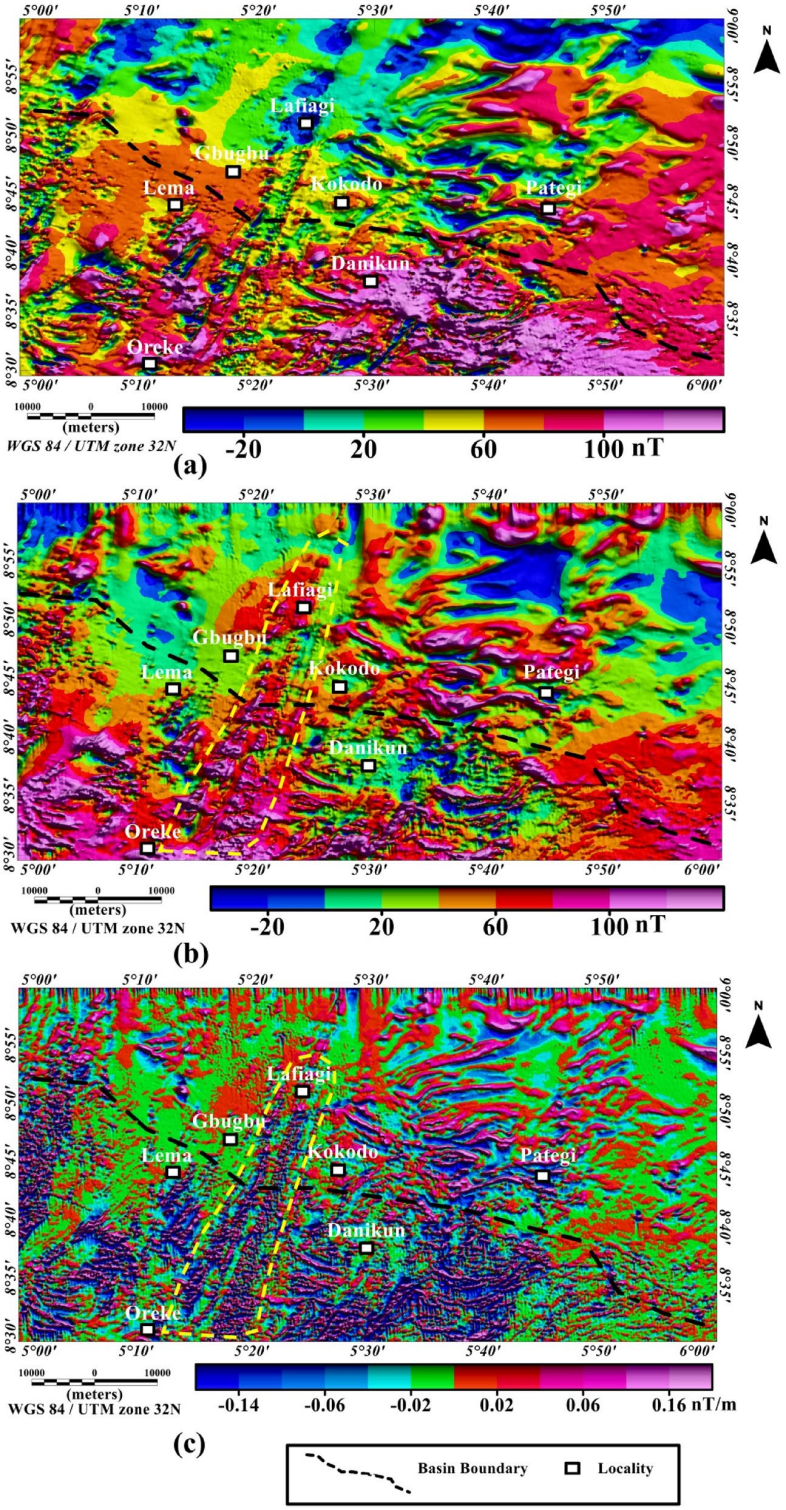
(**a**) Color-shaded residual magnetic intensity anomaly data, with color shading highlighting linear structural features especially at the central part of the study area. Note, at low-latitude regions such as the study area, magnetic sources are usually characterized by magnetic low anomalies. (**b**) Color-shaded reduced-to-pole (RTP) map computed from the residual total magnetic intensity data of the study area. The color shading highlights linear structural features which are shown in areas represented within the yellow dashed lines on the RTP map. (**c**) Color-shade first vertical derivative (FVD) map of the study area produced from the reduced-to-pole (RTP) residual magnetic intensity anomaly data. The RTP-FVD map reveals major linear structural features which are shown in areas represented within the yellow dashed lines on the map. The figures were produced using Oasis Montaj Software version 8.3.3 (Website: https://www.seequent.com/).

The residual magnetic intensity (RMI) map (Fig. 3a) reveal magnetic highs within the central and northern portions of the study area and magnetic lows mostly in the sourthern portion of the RMI map. In most cases, at low-latitude regions such as the study area, sources with positive magnetic susceptibility differences are usually characterized by magnetic lows on the RMI map. The reduced-to-pole (RTP) data computed from the RMI data of the study area is shown in Fig. 3b as color shaded-relief map. The RTP field ranges from − 20 to 100 nT, emphasizing high magnetic signature of the basement complex terrain (see central and southern portions of Fig. 3b). In the northern (around Dalada), northeastern and northwestern parts of the RTP map, prominents low RTP anomalies which are featureless are observed on the map (Fig. 3b). These featureless character (magnetic low anomalies) characterized by large wavelengths are attributed to sedimentary basin because the rocks within the northern (around Dalada), northeastern and northwestern parts of the RTP maps are mainly Cretaceous sediments and alluvial deposits and they are expected to produce weak anomalies. This is because anomalies resulting from non-igneous magnetic sources within sediments are normally much weaker than those resulting from igneous and metamorphic basement rocks that usually contain higher volume of magnetic minerals^26^. In justification, recent aeromagnetic investigation of the Middle Niger Basin by Ref.^18^ revealed that the basin is characterized by the absence of volcanic rocks. There is a regional trend (long wavelength) increase in the field northerly part of the RTP map. In the area between Lade, Lema and Pategi, anomalies with intermediate wavelengths are observed, which revealed the transition from sedimentary basin (in the north) to the basement complex terrain on the southern parts of the study area. There is a concentration of short wavelengths in the southern part of the RTP map, which are associated with structural features within the Archean basement complex terrain.

### First vertical derivative map

Much insight into shallow subsurface structural features of the study area comes from the visual investigation of prominent magnetic signatures on the first vertical derivative (FVD) map (Fig. 3c) produced from the reduced-to-pole residual magnetic intensity anomaly data. The RTP-FVD map (Fig. 3c) was utilized to reveal the shallowest geologic magnetic sources within the study area. Existence of major magnetic gradients within the studied region marked with yellow dashed lines on the RTP-FVD map closely follows the prominent Ifewara shear zone expression on the geological map in Fig. 1. The RTP-FVD map suggests that the major magnetic gradients are related to the Ifewara shear zone in the crystalline basement with the shear zone continuing underneath the Middle Niger Basin. The presence of these major magnetic gradients on the RTP-FVD map implies that the Ifewara shear zone has shallow manifestation; since the FVD transformation enhances near-surface structural features. In the southern part of Fig. 3c, dominant high-frequency anomalies are observed, which are associated with structures within the basement. In contrast, few anomalies are observed in the northern part of the RTP-FVD map, which are associated with the presence of intercalations of magnetic levels in the sediments such as Ironstone beddings within the Middle Niger Basin.

### Magnetic structural and depth estimation maps

**Figure 4:**
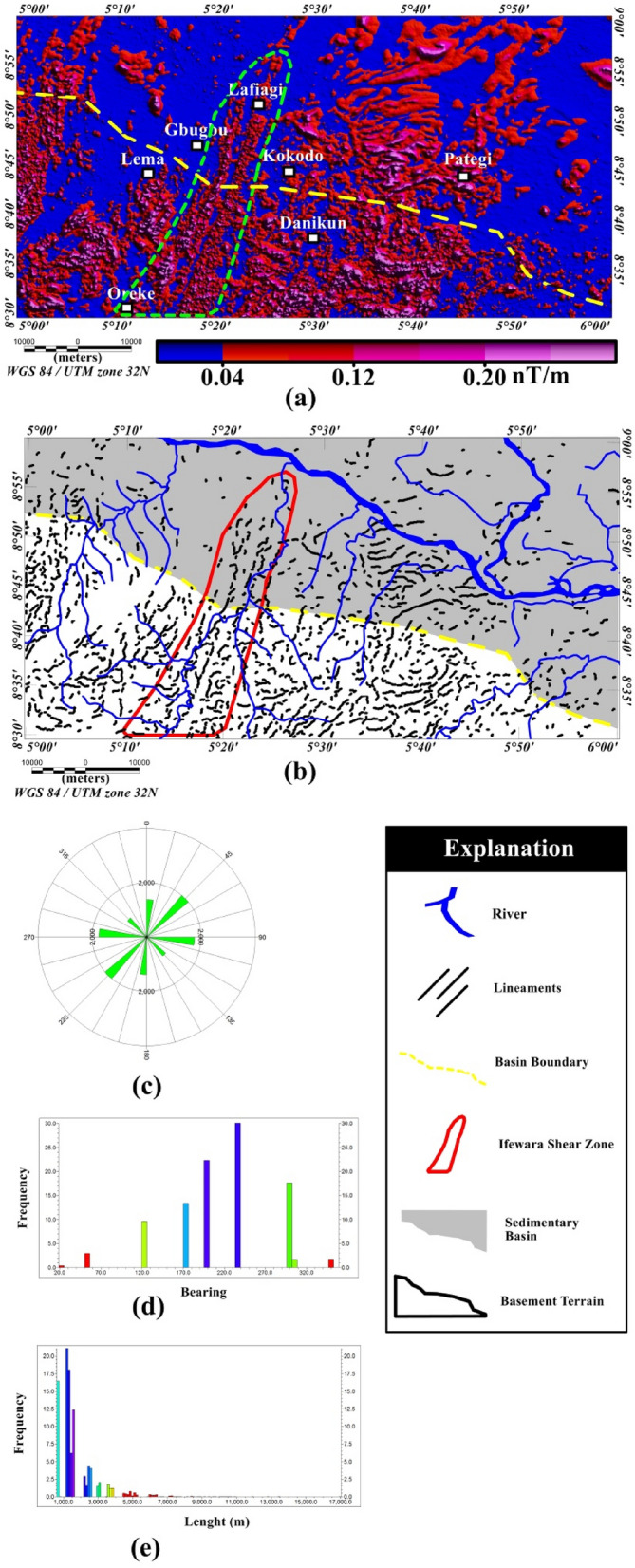
(**a**) Color-shaded total gradient map of Lafiagi and Pategi region produced from the residual magnetic intensity anomaly data. Note the full extent of Ifewara fault on Fig. 5a specified as within the thick green polygon. (**b**) Produced magnetic structural map of the region computed from the identified maxima of the total gradient amplitude method. (**c**) Rose-diagram computed from the maxima of the total gradient amplitude, showing that the magnetic lineaments are mainly oriented in the E–W, NW–SE, N–S and NE–SW directions. (**d**) Bearing–frequency distributions for each magnetic lineament dataset. (**e**) Length–frequency distributions for each magnetic lineament dataset. (**a,b**) Were produced using Oasis Montaj Software version 8.3.3 (Website: https://www.seequent.com/).

The amplitude of the total gradient (TG) map is presented in Fig. 4a, highlighting in part the subsurface structural anomalies with mainly NE–SW and E–W trends. The map depicts higher values of TG amplitude of the residual magnetic intensity anomaly data with amplitude beyond 0.20 nT/m. The values were observed mainly in the regions marked with dashed green lines on the TG map (see Fig. 4a) of the study area. The dashed green region and other locations with high values of TG amplitude are mainly within the crystalline basement complex and are of strong magnetic areas. This suggests that the locations have substantial magnetic susceptibility differences which in turn generate identifiable anomalies on the TG map. In order to depict the lateral extent and locations of lineaments revealed on the TG map, maxima of the TG amplitude were extracted to produce the magnetic structural map (Fig. 4b) of the study area. The map reveals subsurface geometry of the study area, which is characterized by TG lineaments (maxima of the TG amplitude) with different trends. A key observation in the magnetic structural map is the prominent NNE-trending TG lineaments which are concentrated within a zone shown by red color polygon in Fig. 4b. The zone passes through Oreke and Lafiagi and extends to the south and north of the study area. The zone is interpreted as the Ifewara shear zone which is the southern extension of the NNE-trending Kalangai—Zungeru shear zone associated with gold mineralization^17,22^. This major shear zone has been documented as Pan-African crustal sutures and loci of economic mineral deposits ^17,27–29^. The prominent Ifewara shear zone is associated with quartzite ridges within the southern part of the study area and terminates in Oreke. The subsurface evidence of the shear zone which is characterized by lineaments within the zone is now revealed by the magnetic structural map presented herein as a northern elongation of several kilometers of the faults which are associated with quartzite ridges.

**Figure 5:**
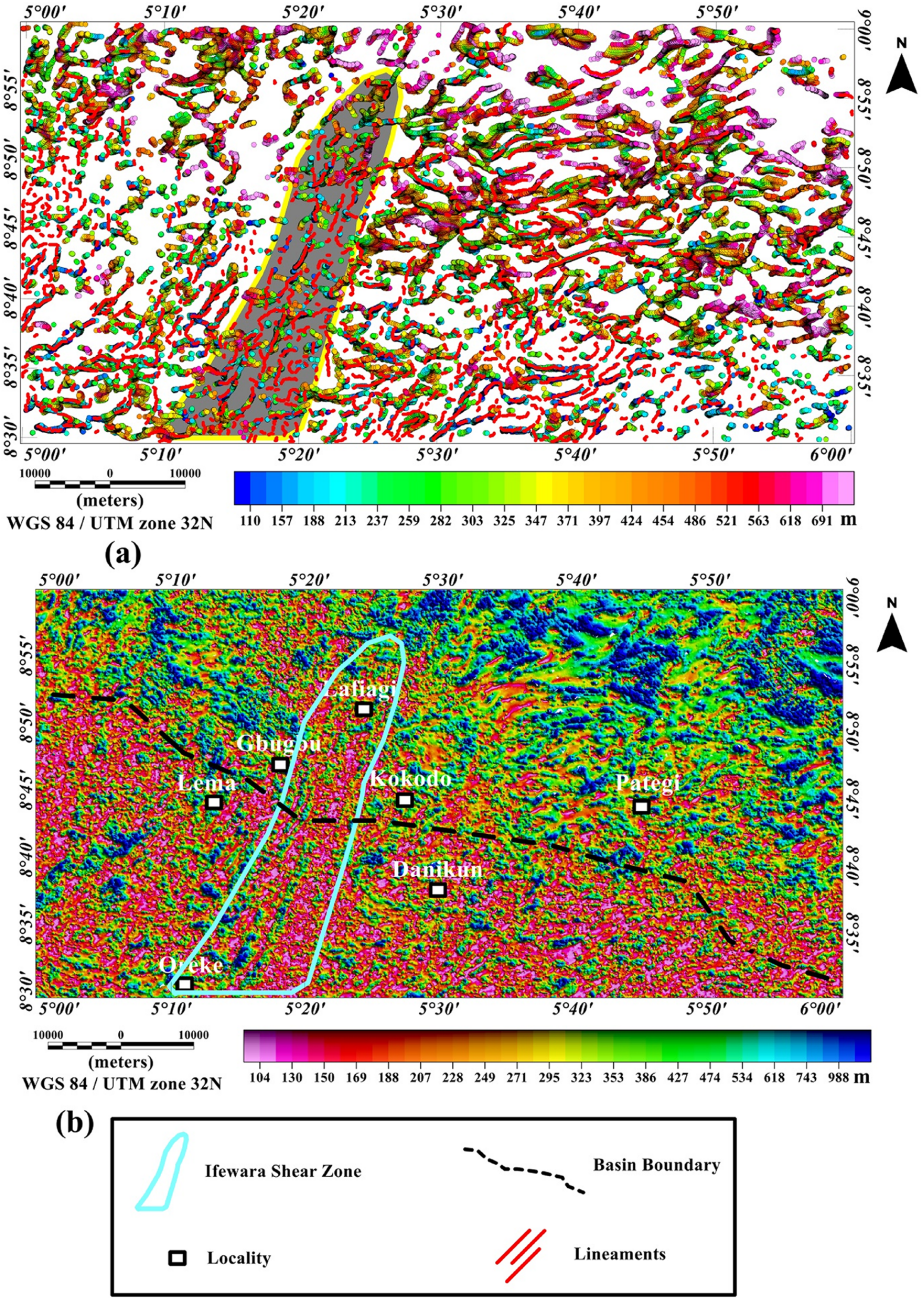
(**a**) 3-D Euler deconvolution depth map generated for SI = 1 using residual magnetic intensity anomaly data of the study area, superimposed with the extracted maxima of the total gradient amplitude in Fig. 4b. Note the 3-D Euler depth solution clusters along the Ifewara shear zone (enclosed in yellow polygon with brown background) and dominant E–W trending 3-D Euler depth solution clusters attributed to pre-Pan-African orogeny are observed in the eastern part of the map. (**b**) Color-shaded source parameter imaging (SPI) depth map of the study area produced from the application of the SPI method on residual magnetic intensity anomaly data of the southern border of the Middle Niger Basin. The blue color polygon indicates the extent of the Ifewara shear zone. The surface from which depths are measured is 80 m which is the sensor mean terrain clearance of the aeromagnetic survey. The figures were produced using Oasis Montaj Software version 8.3.3 (Website: https://www.seequent.com/).

Rose diagram (Fig. 4c), bearing histogram (Fig. 4d) and length histogram (Fig. 4e) were generated from the extracted maxima of the TG amplitude. Visual investigation of the rose diagram (Fig. 4c) facilitated the division of the study area into two distinct structural domains based on trends of magnetic lineaments which provide vital information regarding regional structural features; including the two deformational events within the southern border of the Middle Niger Basin area discussed hereunder. The E–W structural trends show an early deformation and constitute an old fracture system attributed to earlier pre-Pan-African orogeny. The later Pan-African orogeny event (600 Ma) produced the dominant NW–SE, NNE–SSW and NE–SW trends shown on the rose diagram^15^. Additionally, the depths to the top of magnetic structures within the study area are revealed on the 3-D Euler deconvolution map shown in Fig. 5a, with depth values ranging from 110 to 691 m with an average value of 400 m. The extracted maxima of the total gradient map (Fig. 4a) which is shown on the magnetic structural map (Fig. 4b) are overlaid on the 3-D Euler deconvolution (see Fig. 5a) for better understanding of the depths to the top of the delineated TG-lineaments. The 3-D Euler deconvolution clustering depth solutions matched very well with the TG-lneaments (see Fig. 5a) and most of the mapped lineaments fall in the depth range of 110 to 691 m in Fig. 5a. The 3-D Euler decovolution map (Fig. 5a) reveals dominant E–W trending lineaments in the eastern part of the study area. These structural trends show an early pre-Pan-African deformation, which is completely obliterated by the later Pan-African deformational event which produced structural trends such as NW–SE, NE–SW and NNE–SSW in other parts of the study area. Furthermore, source parameter imaging (SPI) map was produced (Fig. 5b) to augment and supplement the initial depth information provided by the 3-D Euler deconvolution depth map (Fig. 5a). The SPI map reveals depth solutions between 104 to 988 m with an average value of 546 m. The SPI map (Fig. 5b) provides information about geometry of the substrate and buried structures within the study area. The prominent Ifewara shear zone shown on the magnetic structural map (Fig. 4b) is well revealed by the SPI depth map which is shown in the blue color polygon in Fig. 5b. It was observed that the depth values to the top of shallow structures within the shear zone vary from 104 to 150 m.

**Figure 6:**
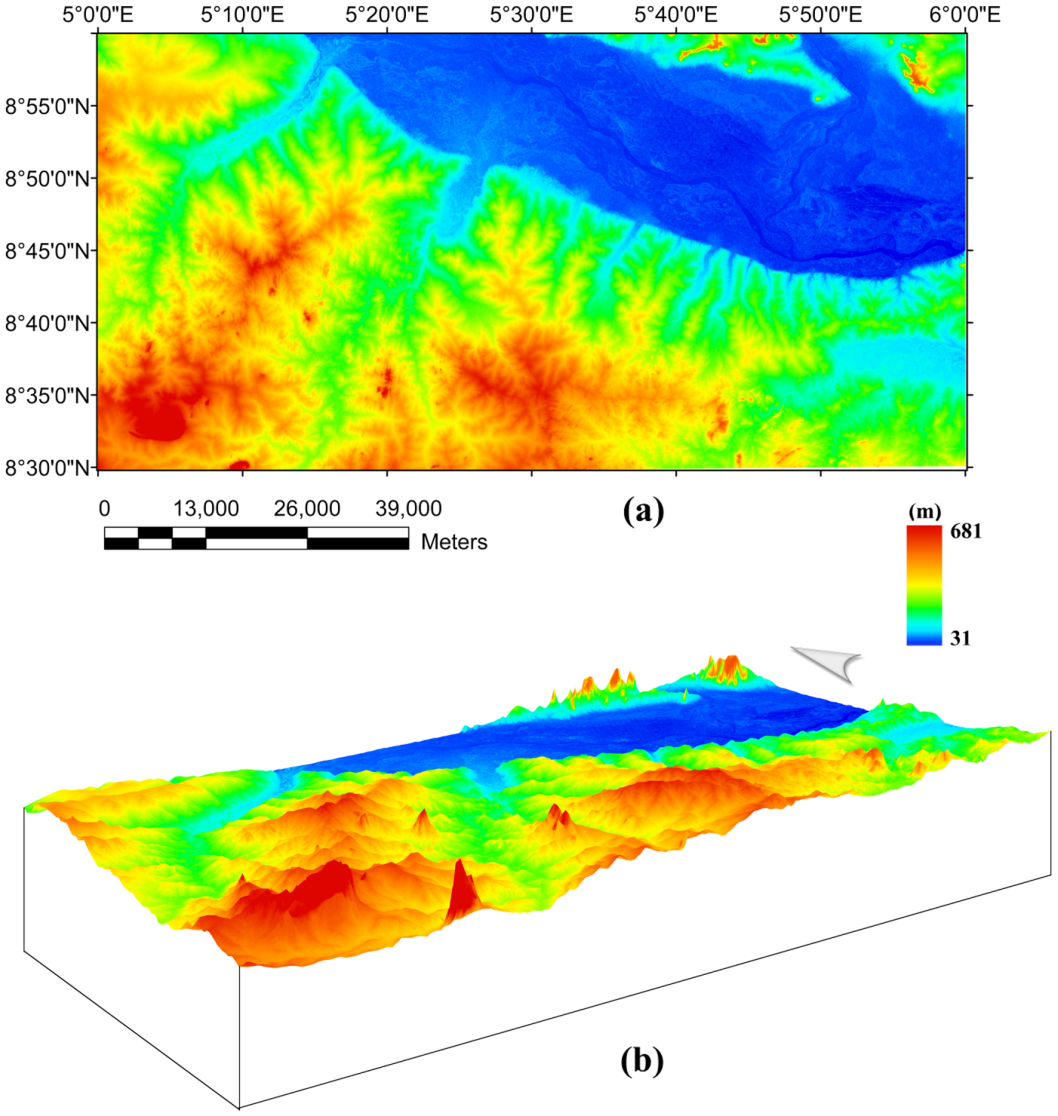
Digital elevation model (DEM) of southern border of the Middle Niger Basin produced from the Shuttle Radar Topographic Mission data. (**A**) 2D map view DEM of study area showing River Niger and its valley network. (**B**) 3D map view DEM of study area showing prominent topographic features. Shuttle Radar Topography Mission image courtesy of the U.S. Geological Survey. The figure was produced using ArcMap Software version 10.1 (Website: https://desktop.arcgis.com/en/arcmap/).

### Morpho-structural interpretation

**Figure 7:**
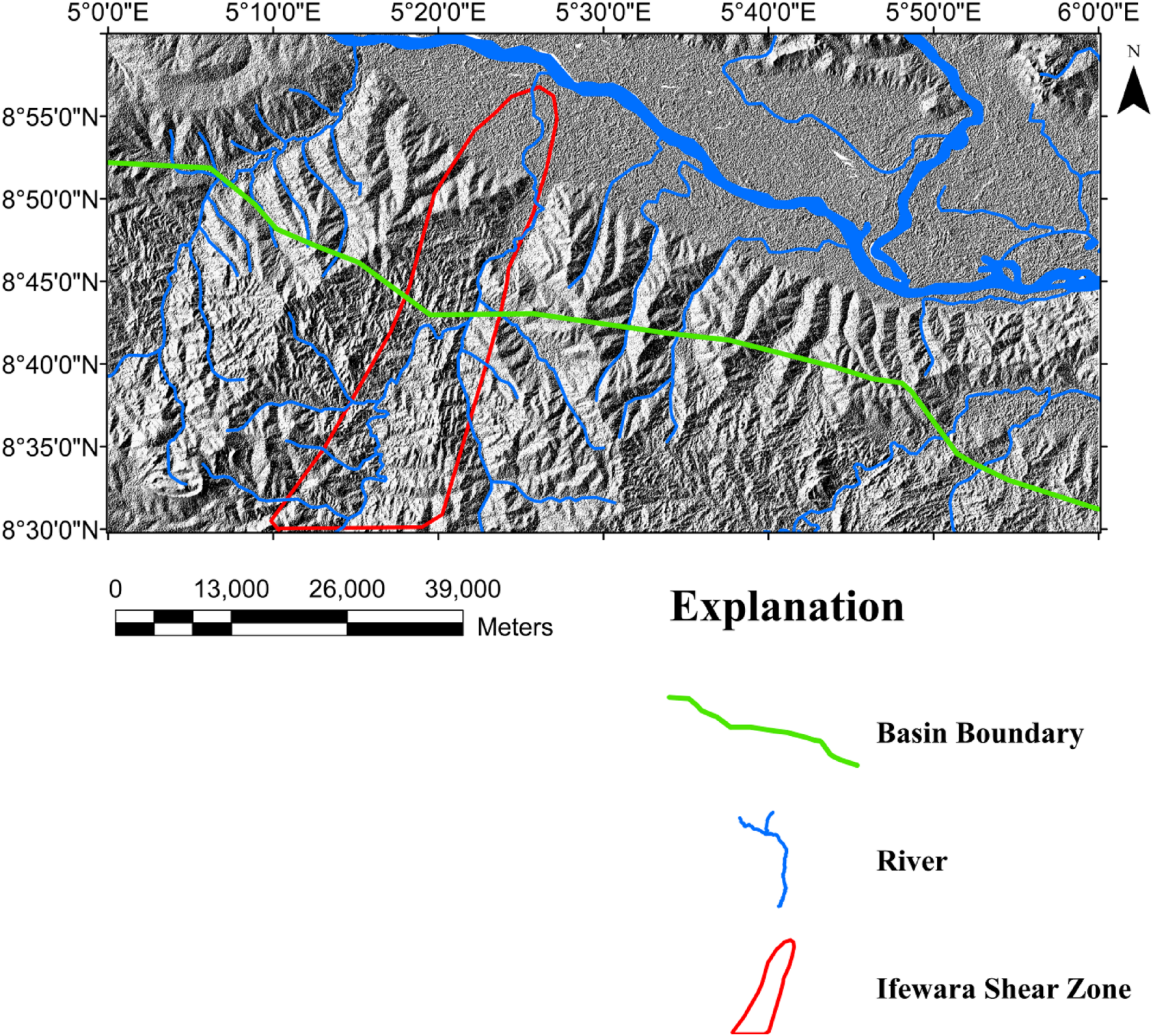
Multi-illumination shaded-relief map of the study area generated from the Shuttle Radar Topography Mission data of southern border of the Middle Niger Basin. The map is produced from a multidirectional hill-shading technique illuminating the terrain from various azimuths (225, 270, 315, and 360 degrees azimuth) at 45 degrees light angle. The parameter values of the LINE module of the PCI Geomatica software utilized for the automatic lineament extraction procedure are shown in Supplementary Table S1. Shuttle Radar Topography Mission image courtesy of the U.S. Geological Survey. The figure was produced using ArcMap Software version 10.8 (Website: https://desktop.arcgis.com/en/arcmap/).

**Figure 8:**
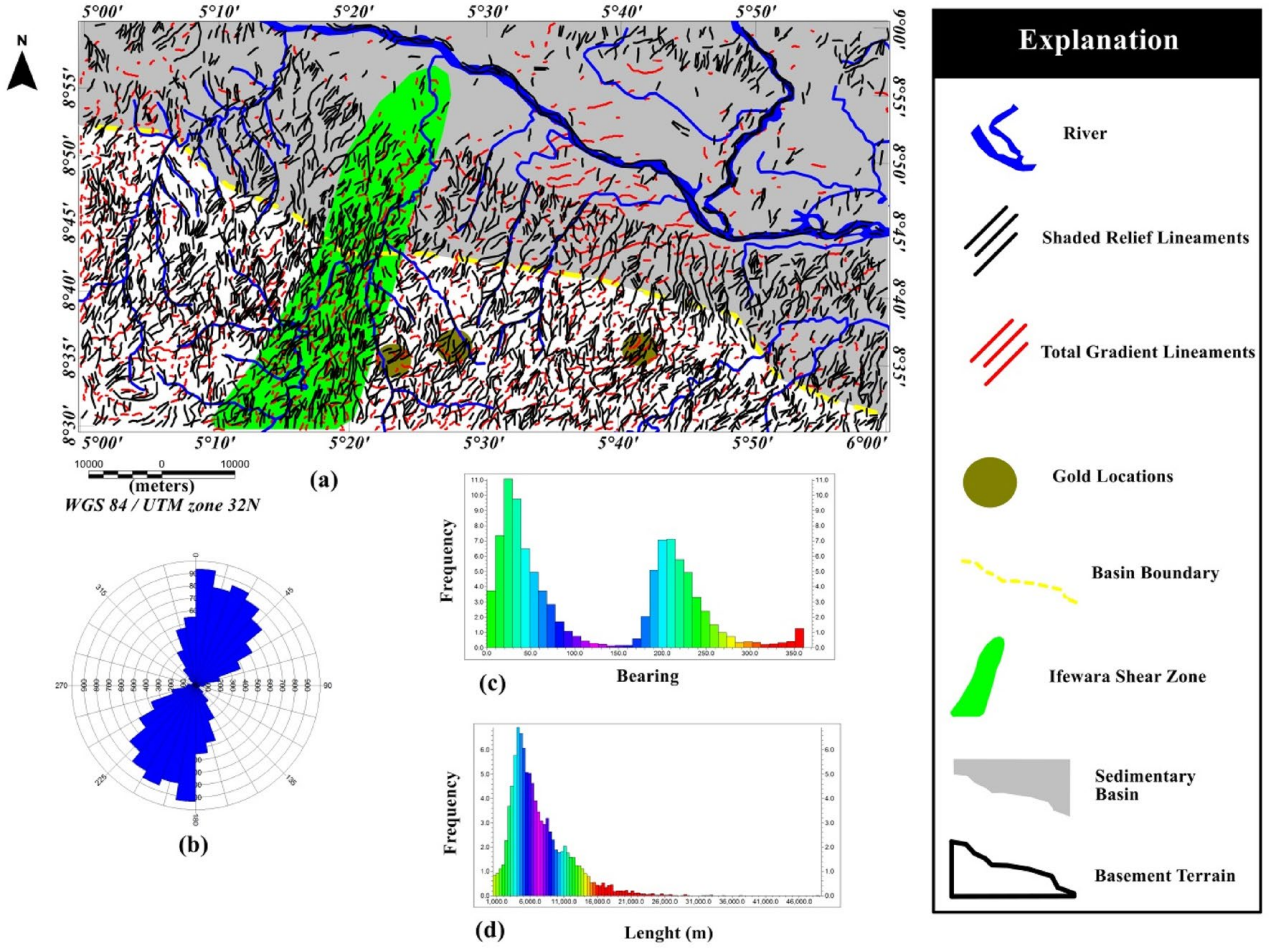
(**a**) Composite structural map of southern border of the Middle Niger Basin produced from the combined analysis and interpretation of SRTM and high resolution aeromagnetic anomaly data. The map depicts good information about surface and subsurface lineament distribution within the study area. Surface lineaments were produced from the multi-illumination shaded-relief map of the southern border of the Middle Niger Basin using a 45 degrees light angle at azimuths 225, 270, 315, and 360 degrees. (**b**) Rose-diagram computed from the automatic extracted surface lineaments, showing that the lineaments are mainly oriented in the N–S, NNE–SSW, NE–SW directions, which provide trend patterns of surface lineaments. (**c**) Bearing–frequency distributions for each surface lineament dataset. (**d**) Length–frequency distributions for each surface lineament dataset. Shuttle Radar Topography Mission image courtesy of the U.S. Geological Survey. (**a**) Was produced using Oasis Montaj Software version 8.3.3 (Website: https://www.seequent.com/).

Surface morpho-structural study of the southern border of the Middle Niger Basin was performed to understand the influence of the delineated total gradient lineaments (Fig. 4b) on surface morphology of the study area. In map view, the topography of the study region has been revealed using the 2-D digital elevation model (DEM) (Fig. 6A) and 3-D DEM (Fig. 6B) maps of the Shuttle Radar Topographic Mission data. The relief is between 31 and 681 m above WGS 84 with all major topographic features of the study area clearly revealed on the maps (Fig. 6A,B). Discontinuous elevated topographic features might have formed as part of the reworked older terrain of the Nigerian basement during the multi stage Pan-African orogeny. As a result of subsequent tectonic events, the terrain may have been fragmented into numerous reliefs with shears surrounding hill ranges. The shaded-relief map (Fig. 7) reveals enhanced topographical features within the study area. The Ifewara shear zone constitutes morphological features on the shaded-relief map (Fig. 7) as linear features associated with a linear ridge within the red color polygon in Fig. 7. The shaded-relief map shows that the Ifewara shear zone is not characterized by a single lineament but rather system of faults. The shear zone is marked by red color polygon on the shaded relief map to facilitate easy identification of lineaments within the shear zone. The major topographic features within the Ifewara shear zone coincide with the distributaries of River Niger, with the distributaries exploiting the zones of weakness, in parallel alignment with the NNE-SSW Ifewara shear zone (Fig. 7). This indicates that the Ifewara shear zone controls the drainage pattern of the region and the linear fluvial meander pattern of the distributaries of River Niger. The meander pattern of the distributaries of the Niger River might have been the effect of weathering and erosion. Preferential weathering can take place along the NNE–SSW trending fractures within the Ifewara shear zone, and the river then follow this course. Intersecting fractures might have produced zigzag river traces along which the river jump from one fracture to another. Since it has been established that the Ifewara shear zone is the southern segment of the NNE-trending Kalangai-Zungeru-Ifewara shear zone associated with gold mineralization^17,22^, the results reveal possible concentration of alluvium gold mineralization within the river banks of the River Niger and its distributaries. Automatic lineament extraction technique applied on the shaded-relief map (Fig. 7) resulted in the delineation of surface lineaments presented in the composite structural map (Fig. 8a). The Rose diagram (Fig. 8b) of surface lineaments reveals the general orientation of surface lineaments trend mainly in the N–S, NNE–SSW and NE–SW directions. In addition, the histograms (bearing and length) of surface lineaments (Fig. 8c,d) were also created. The length histogram (Fig. 8d) shows that the SRTM surface lineaments are longer compared to length histogram (Fig. 4e) of total gradient amplitude lineaments. This is because SRTM data has a higher spatial resolution (30 m) than the aeromagnetic data, which leads to the detection of various longer lineaments.

The extracted SRTM surface lineaments and generated subsurface aeromagnetic lineaments shown in Fig. 4b, were combined in Geosoft software to produce the composite structural map of southern border of the Middle Niger Basin (Fig. 8a). The interpretation of the total gradient map is consistent with the SRTM lineaments, which reveals good correlation between surface and subsurface lineaments on the resulting composite structural map (Fig. 8a). This indicates that the deduced surface lineaments coincide with the subsurface structural features and reflect real continuity of surface lineaments at depth. Present day topographic lineaments correlating with subsurface magnetic structural features imply that the relation between Pan African Orogeny aged structures with present day relief is obvious in the study area, especially within the basement terrain. However, the lack of correspondence of the older (pre-Pan-African Orogeny) E–W trending magnetic lineaments with topographic lineaments is because the landform is influenced by the later Pan-African Orogeny. Rose diagrams for surface and subsurface lineaments (Figs. 4c and 8b) show lineaments trending in the N–S, NE–SW, NNE–SSW, NW–SE and E–W directions. Additionally, these structural trends suggest two major orogenic events mentioned earlier: (1) The E–W lineament trends constitute the older lineament system attributed to the Eburnean (2.2–2.0 Ga), and (2) The Pan-African orogeny event (600 Ma) which produced structural features with N–S, NNE-SSW and NE–SW trends. Visual investigation of the composite structural map (Fig. 8a) reveals obvious match between the lineaments and gold mineralized zones within the study area. The latter has encouraged the prediction of new zones of possible economic mineralization. Gold occurrence locations matched well with some total gradient (TG) magnetic lineaments on Fig. 8a. Hence, detailed geological mapping and other geophysical methods can be focused on the TG magnetic lineaments within and around the study area for further elucidation.

## Data

### Shuttle radar topography mission data

Shuttle Radar Topography Mission (SRTM) data (n08_e005_1arc_v3.tif) is used in this research to investigate influence of subsurface structural features on the topography of the southern border of the Middle Niger Basin. The SRTM data (courtesy United State Geological Survey) shown in Fig. 6, have a spatial resolution of 1-ARC (approximately 30 m). The data were shaded using a 45 degrees light angle at azimuths of 225, 270, 315, and 360 degrees to highlight topographical structural trends. In addition, the analysis of the SRTM topographic data was very effective when combined with the aeromagnetic interpretations, for integrated surface and subsurface structural investigation.

### Aeromagnetic data

The residual magnetic intensity (RMI) data (sheet numbers 203 and 204) used for this research was acquired from the Nigerian Geological Survey Agency (NGSA). The aeromagnetic survey was carried out by Fugro Airborne Surveys for NGSA between 2004 and 2009. The high resolution aeromagnetic data were collected using the 3X-Scintrex CS3 Cesium Vapour Magnetometer, at a sensor mean terrain clearance of 80 m. The flight lines were flown in NW–SE directions perpendicular to the regional structural trends, with line spacing of 500 m and tie lines of 2000 m interval. The aeromagnetic data were corrected for diurnal variation effects and the International Geomagnetic Reference Field corrections were applied to the data to remove the main component of the geomagnetic field. The resulting RMI data (Fig. 3a) provides preliminary information about magnetic structures within the study area and form the basis for all preceding enhancement techniques applied to the aeromagnetic data. In the case of RMI field, geologic sources that are magnetic would be characterized by magnetic low anomalies. All processing of the RMI data was implemented utilizing commercial software platform Geosoft™. Standard filters were used to enhance the RMI data in order to simplify the interpretation of the magnetic anomalies in relation to their geological sources. The most effective way to enhance the RMI data is to understand the geologic control and suitable filter to achieve quality results. Since the study area is situated within low magnetic latitude, hence enhancement filters suitable for low latitude regions were used. The analysis of the RMI data was achieved using techniques such as the reduction-to-pole (RTP), first vertical derivative, total gradient, 3-D Euler deconvolution and source parameter imaging techniques, with each method having different advantages in various geological situations. Hence, the application of various techniques to the same magnetic data provides better results. The RTP transformation is usually applied to RMI data to reduce polarity effects, which are manifested as a shift of the magnetic anomalies away from the centers of their respective sources. These polarity effects are as a result of the vector nature of the magnetic field measured at non-polar regions. Magnetic field parameters such as inclination: − 6.970 degrees, declination: − 2.341 degrees and total magnetic field: 33,190 nT, at the mid-point of the surveyed region (longitude: 5° 30′ and latitude: 8° 45′) are used for the application of the RTP filter. Additionally, a pseudo-inclination of the magnetic field of − 70 degrees was used to stabilize the result of the RTP filter, which is unstable at low-latitude regions such as the study area. The RTP technique commonly requires an assumption that the total magnetizations of sources are aligned parallel to the ambient magnetic field. The RTP data was used for interpretation with the underlying assumption that the magnetization in the studied region is collinear with the ambient magnetic field. The assumption of no remanent magnetization within the studied region was justified by sedimentary formations within the northern parts of the study area that do not carry much remanence. It is also valid for the southern part of the study area by absence of mafic igneous rocks, which commonly have high remanence. A general outlook on the generalized geological map of Nigeria (Fig. 1) shows that volcanic rocks are not present within the study area.

## Conclusion

The results obtained for the characterization of structural features on the basis of structurally controlled gold mineralization from the combination of aeromagnetic and topographic data has provided guide for future mineral exploration programs within southern border of the Middle Niger Basin. The integration of the results deduced from the application of standard enhancement filters to the residual magnetic intensity (RMI) data allows the establishment of magnetic structural map of the study area. The application of hill-shading technique on the Shuttle Radar Topography Mission (SRTM) data resulted in the production of shaded-relief map of the region. The shaded-relief map reveals enhanced topographical features within the study area and the map was utilized for the automatic extraction of lineaments which resulted in the establishment of surface lineament map of the region. Hence, we have produced a composite structural map from the integration of results deduced from the RMI and SRTM imagery. The composite structural map unravels the structural architecture of the region, revealing two sets of lineaments that most probably relate to Eburnean and Pan-African events. The deduced surface and surface lineaments on the composite structural map correlate very well with known gold mineralization locations. The intersections of NE–SW and NW–SE trending lineaments, which are associated with the known gold locations on the composite structural map, have the highest probability for structurally controlled gold mineralization. In map view, the spatial dispositions of River Niger valleys by the Ifewara shear zone have been revealed on the produced shaded-relief map of the region. The Ifewara shear zone coincides with the valleys of the River Niger, which have significant implication for alluvium gold mineralization.

## Supplementary Information

Supplementary Information.

## Data Availability

The Shuttle Radar Topography Mission data used for this study is publicly accessible and can be downloaded from the US Geological Survey (USGS) website. However, the high resolution aeromagnetic data is not publicly available, but can be acquired from the Nigeria Geological Survey Agency.
