# Supplementary material for: Aeromagnetic and digital elevation model constraints on the structural framework of southern margin of the Middle Niger Basin, Nigeria
Source: Sci Rep. 2021 Nov 4;11:21646. doi: 10.1038/s41598-021-00829-y (PMC8569153; doi:10.1038/s41598-021-00829-y)
Supplement: Supplementary file 1 — Supplementary Information. [file 41598_2021_829_MOESM1_ESM.docx]

**Supporting Information**

**Aeromagnetic and digital elevation model constraints on the structural framework of southern margin of the Middle Niger Basin, Nigeria**

Naheem Banji Salawu^1, 2^

1. BS Geophysical and Consultancy Ltd. Nigeria.

2. Department of Geophysics, Federal University Oye-Ekiti, Oye-Ekiti, Nigeria.

**Email Address: salawubanji@yahoo.com**

**Supplementary Table S1:** The utilized parameters for automatic lineament extraction procedure. **Abbreviations**: RADI – Filter Radius (Pixels), GTHR – Edge Gradient Threshold, LTHR – Curve Length Threshold (Pixels), FTHR – Line Fitting Error Threshold (Pixels), ATHR– Angular Difference Threshold (Degrees), DTHR– Linking Distance Threshold (Pixels).

| **Parameter** RADI GTHR LTHR FTHR ATHR DTHR |
| --- |
| **Values**  10 100 30 3 30 20 |
